# Evaluation of the Effect of CYP2D6 Genotypes on Tramadol and *O*-Desmethyltramadol Pharmacokinetic Profiles in a Korean Population Using Physiologically-Based Pharmacokinetic Modeling

**DOI:** 10.3390/pharmaceutics11110618

**Published:** 2019-11-17

**Authors:** Hyeon-Cheol Jeong, Soo Hyeon Bae, Jung-Woo Bae, Sooyeun Lee, Anhye Kim, Yoojeong Jang, Kwang-Hee Shin

**Affiliations:** 1 College of Pharmacy, Research Institute of Pharmaceutical Sciences, Kyungpook National University, Daegu 41566, Korea; houkiboshi01@knu.ac.kr (H.-C.J.); kersy@daum.net (Y.J.); 2 Korea Institute of Radiological & Medical Sciences, Seoul 01812, Korea; shbae@kirams.re.kr; 3 College of Pharmacy, Keimyung University, Daegu 42601, Korea; jwbae11@kmu.ac.kr (J.-W.B.); sylee21@kmu.ac.kr (S.L.); 4 Department of Clinical Pharmacology and Therapeutics, CHA Bundang Medical Center, CHA University, Seongnam 13496, Korea; ahkim1@cha.ac.kr

**Keywords:** CYP2D6, *O*-desmethyltramadol, pharmacokinetics, physiologically-based pharmacokinetics, tramadol

## Abstract

Tramadol is a μ-opioid receptor agonist and a monoamine reuptake inhibitor. *O*-desmethyltramadol (M1), the major active metabolite of tramadol, is produced by CYP2D6. A physiologically-based pharmacokinetic model was developed to predict changes in time-concentration profiles for tramadol and M1 according to dosage and CYP2D6 genotypes in the Korean population. Parallel artificial membrane permeation assay was performed to determine tramadol permeability, and the metabolic clearance of M1 was determined using human liver microsomes. Clinical study data were used to develop the model. Other physicochemical and pharmacokinetic parameters were obtained from the literature. Simulations for plasma concentrations of tramadol and M1 (after 100 mg tramadol was administered five times at 12-h intervals) were based on a total of 1000 virtual healthy Koreans using SimCYP^®^ simulator. Geometric mean ratios (90% confidence intervals) (predicted/observed) for maximum plasma concentration at steady-state (C_max,ss_) and area under the curve at steady-state (AUC_last,ss_) were 0.79 (0.69–0.91) and 1.04 (0.85–1.28) for tramadol, and 0.63 (0.51–0.79) and 0.67 (0.54–0.84) for M1, respectively. The predicted time–concentration profiles of tramadol fitted well to observed profiles and those of M1 showed under-prediction. The developed model could be applied to predict concentration-dependent toxicities according to CYP2D6 genotypes and also, CYP2D6-related drug interactions.

## 1. Introduction

Tramadol is an orally available, centrally acting, weak-opioid analgesic drug [1]. The anti-nociceptive effect of tramadol is due to two mechanisms: an opioid mechanism, and a non-opioid mechanism [2]. Tramadol acts as a μ-opioid receptor agonist, like traditional opioids. It also has analgesic effects by inhibiting reuptake of monoamine neurotransmitters, such as 5-hydroxytryptamine (5-HT) and noradrenaline [3,4]. These mechanisms lead to reduced pain signal conduction and analgesic effects. Tramadol is a racemate, and analgesic mechanisms differ depending on the isomer: (−)-tramadol exhibited ≈ 10-fold higher noradrenaline reuptake inhibitory activity than (+)-tramadol, and (+)-tramadol showed ≈ 4-fold higher 5-HT reuptake inhibitory activity than (−)-tramadol [5].

Tramadol is predominantly metabolized in the liver. Approximately 10%–30% of administered tramadol is excreted, unmetabolized, in the urine. The well-known metabolic pathway of tramadol is divided into three major pathways: *O*-desmethyltramadol (M1) is produced by cytochrome P450 (CYP) 2D6, and *N*-desmethyltramadol (M2) is produced by CYP2B6 and CYP3A4. These metabolites are converted to either *N*,*O*-didesmethyltramadol (M5) and other inactive metabolites by CYP2D6, CYP2B6, and CYP3A4, or converted to glucuronides by UDP-glucuronosyltransferase (UGT) 1A8 and UGT2B7 [6]. M1, a major active metabolite of tramadol, has about 700-fold higher affinity for μ-opioid receptors than tramadol [7]. M5 also has 24-fold higher affinity for μ-opioid receptors than tramadol [3]. Tramadol is mainly considered to inhibit monoamine reuptake, while M1 and M5 bind to μ-opioid receptors and exhibit analgesic effects. Thus, genetic polymorphism of CYP2D6 could have an effect on the risk of adverse events during tramadol administration [4].

A physiologically-based pharmacokinetic (PBPK) approach is a bottom-up approach that requires data about the physicochemical properties and pharmacokinetic (PK) parameters (i.e., absorption, distribution, metabolism, and excretion; ADME) of the target drug [8,9]. In addition, the PBPK model considers bodyweight, height, organ volume, blood flow, and inter-individual variation for metabolizing enzymes and transporters [10]. Therefore, the PBPK model can be used to predict plasma concentration–time profiles more closely than traditional compartmental PK models [11]. In this regard, PBPK modeling can predict human PK profiles using in vitro or preclinical study data from drug development. Further, such modeling is also used to investigate the interaction potential with other drugs or food, and to predict PK profiles in special populations, such as pregnant women, geriatric patients, or children [9,12].

Studies predicting the PK profile of tramadol using PK modeling have been reported previously. Many articles used a population PK approach with nonlinear mixed-effects modeling (NONMEM), however a PBPK approach was rarely used to predict the PK profile of tramadol [13,14,15]. When tramadol was administered, M1 also had impact on efficacy and toxicity [16]. Therefore, M1 should be integrated for PBPK model of tramadol for better interpretation.

The aims of this study were to develop a PBPK model that could predict the concentration–time profiles of tramadol and M1 in Koreans, and to investigate effects of the CYP2D6 genotype on PK profiles at routinely administered doses. The developed PBPK model was applied to predict the effects of CYP2D6 genotype and tramadol dosage on the plasma concentration profiles of tramadol and M1 in a healthy Korean population.

## 2. Materials and Methods

### 2.1. Clinical Study Design

The clinical study was approved by the Institutional Review Board of Keimyung University (Deagu, Republic of Korea, approval number: 40525-201509-BR-70-02, 23 February 2016) and Kyungpook National University Hospital (Daegu, Republic of Korea, approval number of clinical trial: 2016-08-005, 24 August 2016) and carried out at the Kyungpook National University Hospital Clinical Trial Center (Daegu, Republic of Korea). A total of 23 subjects participated who voluntarily agreed to take part in the clinical study and signed their informed consent. Subject characteristics are presented in Table 1. All subjects received a 100-mg tramadol hydrochloride tablet (Tridol^®^ extended-release (ER); Yuhan Pharmaceutical, Seoul, Korea) five times at 12-h intervals. Whole blood was collected in an anticoagulant tube at pre-dose, and at 0.5, 1, 1.5, 2, 2.5, 4, 6, 8, 10, 12, 24, 48, and 72 h after administration. The obtained whole blood was used for CYP2D6 genotyping, and the plasma was separated for determination of tramadol and M1 [17].

### 2.2. CYP2D6 Genotyping

The determination of CYP2D6 genotype was performed for CYP2D6*2 (normal function), CYP2D6*5 (no function), and CYP2D6*10 (decreased function). Genotyping for CYP2D6*2 and CYP2D6*10 was performed using pyrosequencing. CYP2D6*5 was sequenced by long polymerase chain reaction (PCR) because of deletion of a specific sequence. Pyrosequencing was performed using Pyromark Q96 ID and Pyromark Gold Q96 reagents (Qiagen, Hilden, Germany). The conditions of PCR (total 35 cycles) were: denaturation (94 °C for 30 s), annealing (56 °C for 30 s), and polymerization (72 °C for 30 s). The processes were finished by extension at 72 °C for 5 min. CYP2D6*5 and duplication were determined by long-PCR, as previously described [18,19]. The *CYP2D6* phenotype was determined based on genotype and activity score [20,21,22,23]. CYP2D6*5 was a non-functional allele and homozygous CYP2D6*5 was classified as a poor metabolizer (PM). Homozygous CYP2D6*10 was classified as an intermediate metabolizer (IM).

### 2.3. Determination of Tramadol and O-Desmethyltramadol (M1) Using LC-MS/MS

The obtained whole blood samples were immediately centrifuged at 4 °C, 3000 rpm for 10 min. The isolated plasma samples were transferred to a new microcentrifuge tube and kept at −70 °C until analysis. The plasma samples were completely thawed, then 10 μL of internal standard (tramadol ^13^C-d_3_ for tramadol, and M1-d_6_ for M1) were added to 100 μL samples and mixed briefly. A total of 300 μL of acetonitrile was added, and then samples were mixed thoroughly for 30 s prior to centrifugation at 2500 rpm for 10 min. The organic solvent layer was transferred to a new polypropylene tube and evaporated under nitrogen gas at 40 °C. Methanol 200 μL was added into tubes containing pellets for reconstitution, and 5 μL of reconstituted sample were analyzed. Analyses were carried out on API3200 tandem mass spectrometry system (AB SCIEX, Framingham, MA, USA) equipped with an Agilent 1260 series HPLC system (Agilent Technologies, Santa Clara, CA, USA). Separation of tramadol and M1 was conducted using a Luna C18 column (5.0 μm, 2.0 × 50 mm; Phenomenex, Torrance, CA, USA). Five millimoles ammonium formate and 0.1% formic acid in methanol (A), and 5 mM ammonium formate solution (B), were used for the mobile phase. The used gradient method was as follows: 0–2 min (97%–5% B), 2–4 min (5% B), 4–5 min (5%–97% B), and 5–8 min (97% B). Electrospray ionization-positive ion mode was used for mass detection. The mass transitions (m/z) used were 264.2→58.1 for tramadol, 268.2→58.1 for tramadol internal standard (IS), 250.2→58.2 for M1, and 256.2→64.1 for M1 IS. To obtain pharmacokinetic parameters, non-compartmental analysis (NCA) was performed using Phoenix (Certara Inc., Princeton, NJ, USA).

### 2.4. Parallel Artificial Membrane Permeability Assay (PAMPA)

To determine the permeability of tramadol, a parallel artificial membrane permeability assay (PAMPA) was performed [24]. Gentest™ Pre-coated PAMPA Plate System (Corning, Tewksbury, MA, USA) was used for the permeability assay. All the processes were followed according to the manufacturer’s protocol. Tramadol hydrochloride, dimethyl sulfoxide (DMSO), phosphate-buffered saline (PBS), and acetonitrile were purchased from Sigma-Aldrich (St. Louis, MO, USA). Tramadol stock solution (1 mM) was prepared using 100% DMSO and diluted to 15 μM using PBS (pH 7.4). The PAMPA plate was equilibrated for 30 min at room temperature before performing the permeability assay. PBS 200 μL was dispensed on the acceptor side and 300 μL of working solution was dispensed on the donor side. Incubation was carried out at room temperature for 5 h, and the acceptor and donor side buffers were analyzed using liquid chromatography-tandem mass spectrometry (LC-MS/MS). A total of 12 replicated samples were assayed and mean permeability was calculated and applied to the model.

### 2.5. Assessment of Intrinsic Clearance of M1

For the experiments, *O*-desmethyltramadol HCl (M1), glucose 6-phosphate, glucose 6-phosphate dehydrogenase, MgCl_2_, β-nicotinamide adenine dinucleotide phosphate (NADP), chlorpropamide, Trizma^®^ base, Trizma^®^ hydrochloride, DMSO and formic acid were obtained from Sigma‒Aldrich (St. Louis, MO, USA). To evaluate the intrinsic clearance of M1 by CYPs, metabolic stability studies under NADPH system were conducted in human liver microsoms (HLM) [25,26]. For details, NADPH-generating system (1.3 mM NADP^+^, 3.3 mM glucose 6-phosphate, 3.3 mM MgCl_2_, and 0.4 unit/mL glucose-6-phosphate dehydrogenase) and HLM 0.25 mg/mL were added and preincubated at 37 °C for 5 min. Then, 20 μM M1 was added and reacted at 37 °C for 0, 1, 5, 10, 20, 30, and 40 min, respectively. The total volume of the reaction mixture was 100 μL. After each reaction, the reaction was terminated by addition of 150 μL of acetonitrile containing an internal standard (100 ng/mL chlorpropamide). All experiments were repeated duplicated and the samples were vortexed for 5 min and centrifuged (13,000 rpm, 4 °C) for 15 min. Then, supernatants were injected into LC-MS/MS and M1 were analyzed. The concentration of M1 (20 μM) at 0 min was used to evaluate the metabolism of CYP through the change of drug concentration over time.

### 2.6. Qunatitaion Methods of M1 in In Vitro Experiments Using LC-QTOF

High-performance liquid chromatography (HPLC)-grade acetonitrile and deionized water were obtained from Berdick and Jackson (Muskegon, MI, USA). HLM (50 donors pooled) were purchased from Corning. Analyses were carried out on Sciex QTOF 5600 plus (AB SCIEX, Framingham, MA, USA) equipped with an Agilent 1260 series HPLC system (Agilent Technologies, Santa Clara, CA, USA). For quantitation of M1, the compounds were separated on a Poroshell 120 (4.6 × 50 mm, 2.7 μm; Agilent Technologies) with an isocratic mobile phase consisting of acetonitrile and 0.1% aqueous formic acid (70:30 *v*/*v*) at a flow rate of 0.5 mL/min. The overall run time was 5 min per sample. The column and autosampler temperatures were maintained at 40 °C and 4 °C, respectively. Time-of-flight mass spectrometry analysis was selected in positive ion mode for the sample analysis. The quantitative analytical data were processed using PeakView^®^ (Version 2.2.0; AB SCIEX, Framingham, MA, USA) and MultiQuant^®^ (Version 3.0.2; Framingham, MA, USA), and the formulas C_15_H_23_NO_2_ (M1), and C_10_H_13_ClN_2_O_3_S (chlorpropamide) were used for quantitation.

### 2.7. Development of PBPK Model for Tramadol and M1

PBPK model development was performed using SimCYP^®^ simulator version 18 (Certara, Sheffield, UK). Most of the parameters for tramadol and M1 were entered with reference to the literature. According to previous reports, tramadol was not substrate for P-glycoprotein (P-gp) (ABCB1) and the role of proton-based pumps such as OATP for tramadol permeability was unclear [27,28]. Therefore, in this study, the permeability of tramadol was determined by PAMPA assay. The advanced drug absorption and metabolism model was used to consider the ER formulation, and the dissolution profiles of Tridol^®^ ER 100 mg (Yuhan Pharmaceutical, Seoul, Korea) were applied. The elimination profile for M1 applied the intrinsic clearance by HLM. Kp scalar of tramadol was set to match the observed Vss and the predicted V_ss_ value in the model, and Kp scalar of M1 was obtained from parameter estimation. In clinical study, the V_ss_ of tramadol was calculated to 2.6 L/kg by non-compartmental analysis. That of M1 was not calculated because the exact dose of M1 is unknown. Intrinsic clearance involved in tramadol metabolism was estimated using retrograde model option, and human liver microsomal intrinsic clearance was applied to M1 library. The renal clearance of tramadol and M1 were applied for the predicted value which is the closest to the observed blood concentration–time profile by parameter estimation. The PBPK model was evaluated so that it could effectively predict PK profiles for tramadol and M1 when observed mean plasma concentrations fitted to the predicted plasma concentration–time profile and its 90% confidence interval (CI). The other evaluation criteria were geometric mean ratio for peak plasma concentration at steady-state (C_max,ss_), and area under the plasma concentration–time curve at last observation at steady-state (AUC_last,ss_), and the 90% CI for these values. If the geometric mean ratio and its 90% CI were within the range 0.7–1.43, the model was considered to fit well.

### 2.8. Prediction of Changes in Concentration–Time Profiles for Tramadol and M1 According to CYP2D6 Genotype and Dosing Regimen

The therapeutic range and toxic range of tramadol and M1 were determined by reference to the literature. Because the manufacturer’s recommended acceptable maximum dose of Tridol^®^ ER was 400 mg per day, the tramadol ER tablet was administered to a virtual healthy Korean population at 100 and 200 mg (5 times at 12-h intervals) to simulate the change of concentration–time profiles for tramadol and M1. This simulation assumes linear PK properties for multiple doses of tramadol 100 mg and 200 mg [29]. The effect of CYP2D6 genotype was also simulated for tramadol 100 mg and 200 mg for populations consisting of CYP2D6 poor metabolizers (PM), intermediate metabolizers (IM), extensive metabolizers (EM), and ultra-rapid metabolizers (UM).

## 3. Results

### 3.1. Clinical Study

**Figure 1:**
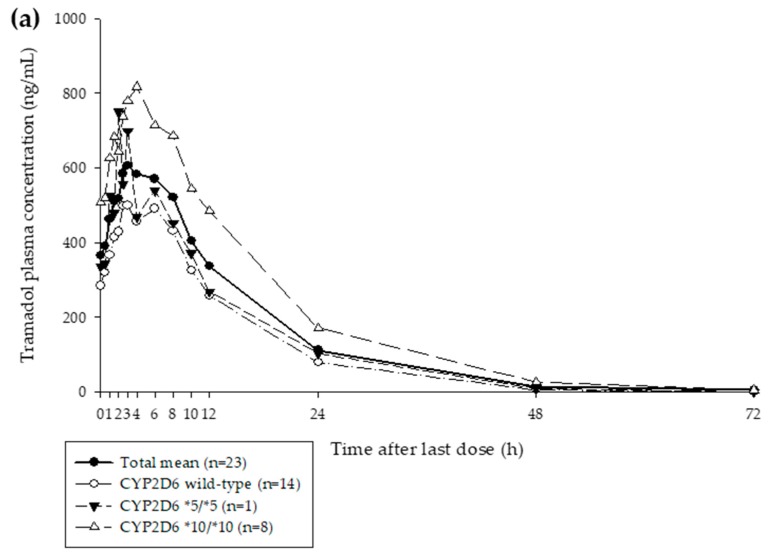

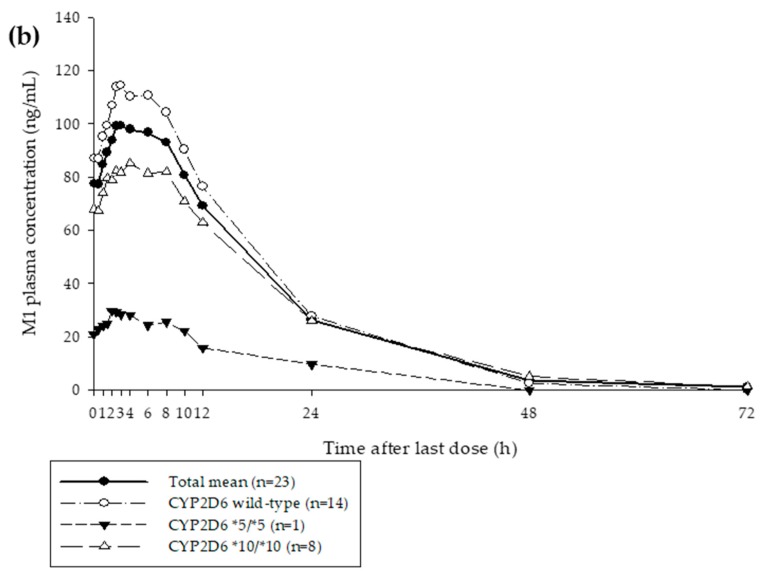
The average plasma concentration–time profiles after five times oral administration (*τ* = 12 h) of 100 mg tramadol for (**a**) tramadol and (**b**) *O*-desmethyltramadol (M1). Solid blue line, average for all subjects (*n* = 23); solid black line, *CYP2D6* wild-type subjects (*n* = 14); short dashed black line, *CYP2D6* *5/*5 subject (*n* = 1); long dashed black line, *CYP2D6* *10/*10 (*n* = 8).

A total of 23 subjects, aged 20–34 years, were enrolled in the clinical study. The average plasma concentration–time profiles for tramadol and M1 are shown in Figure 1. The allele (phenotype) frequencies for *CYP2D6* were: Wild-type (EM, 14 subjects), *10/*10 (IM, 8 subjects), and *5/*5 (PM, 1 subject). 

### 3.2. Metabolism Assay for O-Desmethyltramadol (M1)

We conducted metabolic stability study of M1 and confirmed that M1 was metabolized mainly by CYPs and partially by UGTs. In control sample without NADPH or HLM, more than 96% of tramadol and M1 remained during the incubation time, indicating that the disappearance of tramadol and M1 were mainly caused by CYP enzymes. Metabolic stability of M1 by CYP was assessed using results of the disappearance test for M1. The slope of linear regression was calculated and intrinsic clearance (CL_int_) of the drug in the in vitro microsome system was calculated:

CL_int,mic_ (μL/min/mg protein) = k × V_incubation_/C_incubation_ (1)

where V_incubation_ is incubation volume, C_incubation_ is concentration of microsomal protein.

**Figure 2:**
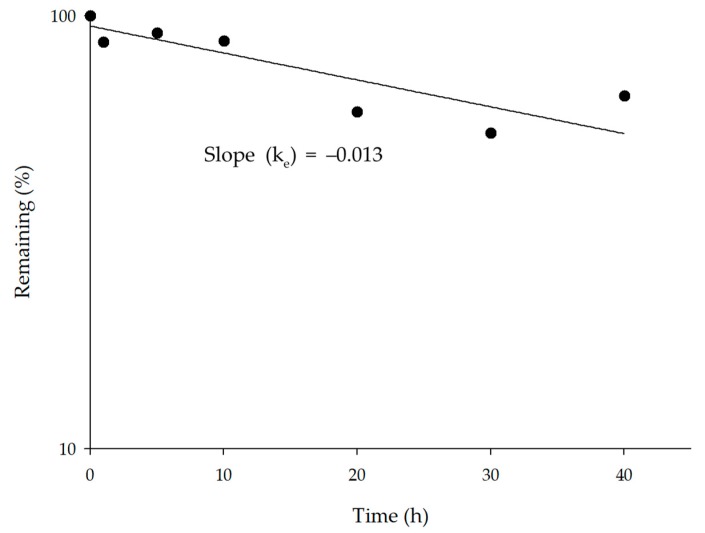
The plot of remaining rate of *O*-desmethyltramadol (M1) after incubation with human liver microsoms (HLM). Each point (obtained by duplicate measurements) represents the mean value. The intrinsic clearance by HLM (CL_int,mic_) was calculated as 52.92 μL/min/mg protein.

To apply the elimination profile to *O*-desmethyltramadol (M1), an HLM assay was performed. The M1 disappearance test showed an HLM intrinsic clearance (CL_int,HLM_) of 52.95 µL/min/mg protein (Figure 2). CL_int,HLM_ for M1 was applied to the M1 PBPK model.

### 3.3. PAMPA Results

Results of the PAMPA assay for 12 tramadol samples diluted to 15 μM showed permeability ranges from 9.14 × 10^−6^ cm/s to 11.5 × 10^−6^ cm/s. The mean ± standard deviation permeability was calculated as 10.4 × 10^−6^ ± 0.056 cm/s. The calculated mean PAMPA permeability was applied to the tramadol PBPK model.

### 3.4. Development of the PBPK Model for Tramadol and M1

**Figure 3:**
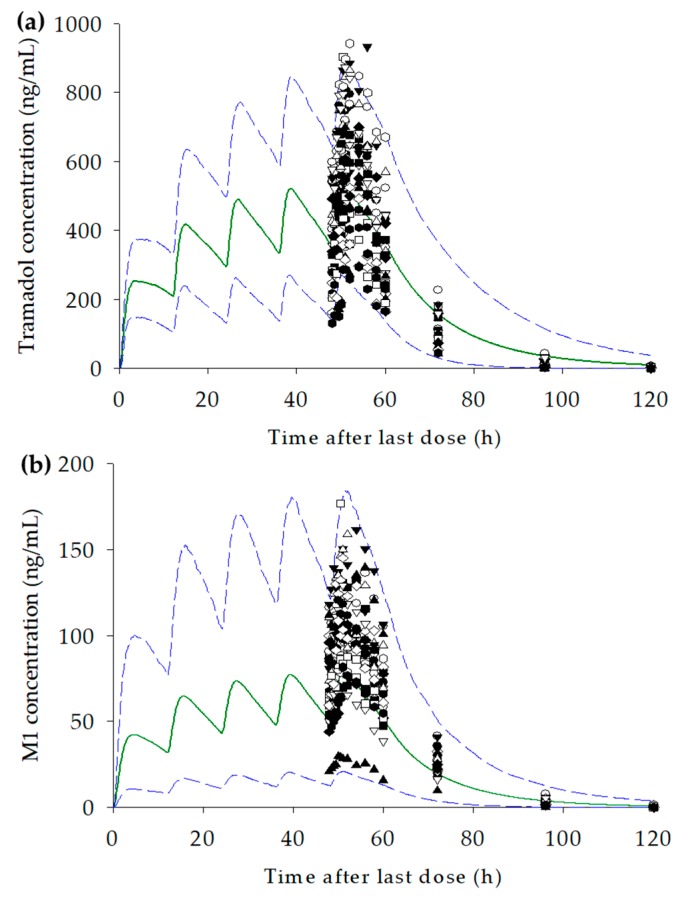
The observed (each symbol, *n* = 23) and simulated mean (solid dark green line) plasma concentration–time profiles after administration of 100 mg tramadol extended-release (ER) tablet twice daily (total five times) for (**a**) tramadol, and (**b**) *O*-desmethyltramadol (M1); blue dashed line represents 5th and 95th percentiles.

The input parameters for tramadol and M1 and demographic characteristics for virtual population in the PBPK model are presented in Table 2 and Table 3. Data for the healthy Korean population were obtained from the Certara repository. Ten virtual trials, including 100 virtual subjects in each virtual trial (total 1000 subjects), were performed for tramadol and M1. In the tramadol model, most of the observation profiles were within the 5th and 95th percentile range, and the predicted mean tramadol concentration in plasma was similar to the observed profile (Figure 3a). The geometric mean ratios of C_max,ss_ and AUC_last,ss_ for tramadol were 0.79 and 1.04, respectively (Table 4). Most observed concentration–time profiles were included in the 5th and 95th percentiles of the predicted concentration–time profiles. In addition, the mean predicted plasma tramadol concentration was well fitted to the observed tramadol concentration. In the concentration–time profiles for M1, most of the observations were also within the 5th and 95th percentile range of the predicted profile (Figure 3b). The range of 90% CI for C_max,ss_ and AUC_last,ss_ were included in the range of 0.7–1.43 (30% range of geometric mean ratio); however, the geometric mean ratio was predicted to be relatively low (Table 4). Both tramadol and M1 were predominantly distributed in the liver. The second most common distribution sites were the spleen (tramadol) and heart (M1; Table 5).

### 3.5. Prediction of Changes in Concentration–Time Profiles for Tramadol and M1 According to CYP2D6 Genotype and Dosage

To investigate the effect of CYP2D6 genotype and dosage on PK profiles, simulations were performed for the administration of 100 and 200 mg of tramadol every 12 h (total 5 times). The tramadol/M1 concentration–time profiles were captured from the pre-dose (0 h) to 120 h. The differences on PK profiles according to CYP2D6 genotypes were assessed in the general Korean population in CYP2D6 groups: PM, IM, EM, and UM. As a result, plasma concentration–time profiles for tramadol were within the therapeutic range in all groups after administration of 100 mg tramadol ER. Predicted plasma M1 concentrations were very low in the PM group (mean C_max,ss_ 0.643 ng/mL) compared to the CYP2D6 IM, EM, and UM groups (mean values 40.93, 83.80, and 126.8 ng/mL, respectively).

**Figure 4:**
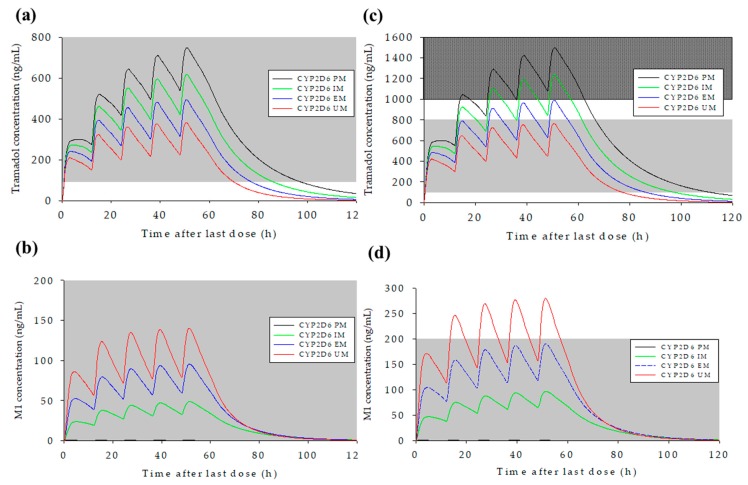
The predicted mean concentration–time profiles after administration of 100 mg and 200 mg tramadol ER tablet twice daily (total five times) for tramadol (**a** and **c**), and *O*-desmethyltramadol (**b** and **d**), respectively. Gray areas in (**a**) and (**c**) represent the therapeutic concentration range (100–800 ng/mL); checked gray area in (**c**) represents the toxic range (above 1000 ng/mL) for tramadol; and the gray area in (**b**) and (**d**) represents the maximum therapeutic range for M1 (up to 200 ng/mL).

The plasma concentration–time profiles for tramadol and M1, and changes in PK parameters, in the various CYP2D6 genotype groups following oral administration of 100 and 200 mg tramadol ER tablet twice daily (total five times) are shown in Figure 4 and Table 6 (the plasma concentration–time profiles for each CYP2D6 phenotype after administration of 100 and 200 mg of tramadol were presented in Supplementary Materials Figures S1 and S2). Following tramadol 100 mg administrations, the C_max,ss_ of tramadol in CYP2D6 PMs reached to toxic range. For CYP2D6 UMs, the C_max,ss_ of M1 exceeded the therapeutic margin (Supplementary Materials Figure S1). Following tramadol 200 mg administrations, the C_max,ss_ of tramadol were reached to the toxic range in all CYP2D6 metabolizer groups. For M1, the C_max,ss_ exceeded the therapeutic margin in the CYP2D6 IMs, EMs, and UMs (Figure S2). In Table 7, observed and predicted C_max,ss_ and AUC_last,ss_ values, and predicted/observed geometric mean ratios are presented. The CYP2D6 UM group was excluded from this table because UM subjects did not exist in the clinical study. In the CYP2D6 EM and IM groups, the predicted/observed geometric mean ratios for C_max,ss_ and AUC_last,ss_ for tramadol satisfied the acceptance criteria (0.7–1.43); however, the tramadol AUC_last,ss_ ratio for the CYP2D6 PM group was overestimated at 1.95. The prediction results for M1 showed that AUC_last,ss_ satisfied the acceptance criteria in the CYP2D6 EM group; however, C_max,ss_ and AUC_last,ss_ values were underestimated in both the CYP2D6 IM and PM groups, where the predicted values were much lower than observed values.

## 4. Discussion

PBPK models for tramadol and M1 were developed. Tramadol plasma concentration–time profiles were well predicted from the proposed model. Prediction results for M1 included values in the 5th to 95th percentiles of most observed plasma concentration–time values, and the predicted mean plasma concentration was also similar to the observed concentration–time profile. However, geometric mean C_max,ss_ and AUC_last,ss_ ratios were under-predicted (0.63 and 0.67 for C_max,ss_ and AUC_last,ss_, respectively). To predict concentration-dependent toxicities, the therapeutic range (100–800 ng/mL for tramadol, and up to 200 ng/mL for M1) and the tramadol toxic range and lethal concentration (>1000 ng/mL, and >2000 ng/mL, respectively) were obtained from the literatures [34,35]. In general, the recommended dose of tramadol is up to 400 mg per day for immediate-release formulations and 300 mg per day for ER formulations [2]. Simulations were performed for 100 and 200 mg with 12-h intervals (5 times) according to CYP2D6 genotypes. After administration of 100 mg of tramadol, the predicted C_max,ss_ of tramadol reached to toxic range in CYP2D6 PMs and exceeded therapeutic range in some IMs, and the predicted C_max,ss_ of M1 exceeded therapeutic margin in CYP2D6 UMs. After tramadol 200 mg administrations, the predicted tramadol C_max,ss_ reached to toxic ranges in all CYP2D6 metabolizer groups, even in some EMs and the predicted M1 C_max,ss_ exceeded the therapeutic margin in CYP2D6 IMs, EMs, and UMs. The concentrations exceeded the therapeutic margins or reached to the toxic range might be related to potential toxicities after tramadol administrations, even though recommended doses of tramadol were administered.

PBPK modeling is useful for predicting PK profiles for rare genotypes in the population. The frequency of CYP2D6 UM in the Korean population has been reported as approximately 1.25% [36]. In the clinical study used for our PBPK model development, there was only one PM subject, and no UM subject was found. The model developed in this study could predict the plasma concentration–time profiles of tramadol and M1 for these two groups. Using the developed model, plasma tramadol/M1 concentration–time profiles for CYP2D6 UM, a very rare genotype in Koreans, were also predicted.

Tramadol inhibits reuptake of 5-HT and norepinephrine. M1 binds to μ-opioid receptors and exhibits analgesic effects. Due to these actions, the side effects of tramadol differ depending on CYP2D6 genotype. In the PM group, a high risk of side effects due to tramadol, such as serotonin syndrome, can be expected; and in the UM group, a high risk of μ-opioid receptor-related side effects, such as respiratory depression, can be expected relative to other CYP2D6 genotypes [16]. In our simulation, the plasma concentrations of tramadol and M1 exceeded the therapeutic concentration range, even after administration of recommended doses. These results suggest that the frequency of concentration-related adverse drug reactions may be reduced by optimizing the dosing regimen according to CYP2D6 genotype of the patient or population.

Tramadol and M1 distribution in each tissue were estimated using the PBPK model, and tramadol and M1 were distributed most to the liver. In cases of fatal intoxication due to tramadol, the highest concentration of tramadol was evident in the liver, after the blood and urine. These distribution characteristics are considered due to the hepatic metabolism of tramadol and its metabolites [37]. The distribution of tramadol to adipose tissue differed from that for M1. Indeed, tramadol is considered to distribute widely to lipid-rich tissues because of its higher affinity for lipids than M1 (logD for tramadol and M1: 1.13 and 0.4, respectively) [38]. Further research is needed about the distribution characteristics of tramadol and M1 to each tissue.

The predicted plasma M1 concentration–time profiles were under-predicted due to a lack of information about distribution and elimination properties. Since M1 is produced by tramadol metabolism, elimination profiles (intrinsic clearance by CYP, renal clearance and additional clearance) of tramadol were adjusted to improve the M1 model; however, there were no significant changes in M1 concentration–time profiles. This might be due to poor distribution of M1 from liver to plasma, or to exaggeration of elimination. For improvement, the M1 model was built using parameter estimation by observed plasma concentration–time profiles as distribution and elimination profiles (tissue–plasma partition coefficient, additional clearance, renal clearance, bile clearance). When estimating several parameters, the predicted plasma M1 concentration–time profiles changed significantly when values of the unbound fraction in incubated microsomes (fu_mic_) and active hepatic scalar were changed. Thus, the plasma M1 concentration–time profile might be greatly influenced by metabolism. More detailed information and parameters for M1 metabolism are needed for more accurate predictions of plasma M1 concentration–time profiles.

Regarding limitations of our study, tramadol is metabolized not only to M1, but also to *N*-desmethyltramadol (M2) by CYP2D6, CYP2B6, and CYP3A4. In accordance with the literature, the toxicity of tramadol and M1 can be determined using M1/M2 ratio [34]. Therefore, an M2 model could improve the predictability of concentration-related adverse drug reactions after tramadol administration. Moreover, organic cation transporter 1 (OCT1) and multidrug resistance protein 1 (MDR1) influence the disposition of tramadol and M1. Significant differences in drug disposition according to OCT1 and MDR1 genotypes have been shown, even in same CYP2D6 phenotype [39,40,41]. Due to lack of information of transporter kinetic parameter for each organ, the transport kinetic parameters for M1 were excluded for the model. For elaborate model prediction, OCT1 and MDR1 genotypes (OCT*1, *2, *3, *4, *5, and *MDR1* C3435T) could be incorporated.

## 5. Conclusions

In summary, our PBPK model for tramadol and M1 was developed and predicted concentration–time profiles after multiple administrations of a tramadol ER formulation in the Korean population. Differences in PK profiles and concentration-dependent toxicities were predicted according to CYP2D6 phenotype and dosage. Most modeling studies of tramadol used a population PK approach, and the literature using PBPK modeling focused on the PK profile of tramadol itself. However, this study developed a model with predictive power for tramadol and M1, the major active metabolite. This model could be applied to predict concentration-dependent toxicity profiles in cases of tramadol overdose or abuse and also, CYP2D6-related drug interactions.

## Figures and Tables

**Table 1:** Demographic characteristics (*n* = 23).

Characteristic	Mean (SD)
Age (years)	24.78 (4.80)
Height (cm)	176.51 (5.64)
Weight (kg)	71.61 (8.87)
CYP2D6 genotypes (no. of subjects)	
Wild-type	14
*5/*5	1
*10/*10	8

SD, standard deviation.

**Table 2:** Input parameters for tramadol and *O*-desmethyltramadol (M1) in the physiologically-based pharmacokinetic (PBPK) model.

Parameters	Tramadol		M1 *	
	Value	Source	Value	Source
***Physicochemical properties and blood binding***				
Molecular weight (g/mol)	263.4	[30]	249.354	[31]
Log *P*	1.35	[30]	2.26	[32]
pKa	9.41 (Monoprotic base)	[30]	9.62 (Monoprotic base)	[32]
fu_p_	0.8	[33]	0.525	Predicted in SimCYP
***Absorption***				
Absorption type	PAMPA	-	n/a	-
P_app_ (×10^−6^ cm/s)	10.2	Experimental data	n/a	-
***Distribution***				
Kp scalar	0.946	Adjusted using V_ss_	0.107	Estimated
V_ss_ (L/kg)	2.6	Observed data	0.628	Estimated
***Elimination***				
CL_int_ (μL/min/pmol or mg protein)	CYP2D6: 0.447; CYP2B6: 0.028; CYP3A4: 0.020	Retrograde model	52.92 (WOMC–HLM)	Experimental data
CL_R_	1.850	Estimated	0.481	Estimated

CL_int_: intrinsic clearance; CL_R_: renal clearance; CYP: cytochrome P450 superfamily; fu_p_: unbound fraction in plasma; HLM: human liver microsomes; Kp: plasma-tissue partition coefficient; PAMPA: parallel artificial membrane permeability assay; P_app_: apparent permeability; V_ss_: volume of distribution in steady-state; WOMC: whole organ metabolic clearance, n/a: not applicable. * Metabolite model does not take account of absorption.

**Table 3:** The demographic characteristics of the participated subjects for virtual Korean population (*n* = 1000).

Parameters	Mean (Range)
Age (years)	28.9 (20.2–40.0)
Height (cm)	166.2 (147.6–188.0)
Weight (kg)	62.3 (42.9–93.9)
The percentage of female	50%

**Table 4:** Observed and simulated pharmacokinetic (PK) parameters for tramadol and *O*-desmethyltramadol (M1) after oral administration of 100 mg tramadol ER tablet twice daily (five times in total).

Parameters	Observed (Range)	Simulated (Range)	Ratio (90% CI)
***Tramadol***			
Geometric mean C_max,ss_ (ng/mL)	643.8; (294.0–942.1)	508.4; (122.1–1226)	0.79; (0.69–0.91)
Geometric mean AUC_last,ss_ (ng/mL·h)	8965; (4127–16,038)	9346; (1217–42,462)	1.04; (0.85–1.28)
***M1***			
Geometric mean C_max,ss_ (ng/mL)	103.8; (29.8–176.7)	65.68; (1.07–368.1)	0.63; (0.51–0.79)
Geometric mean AUC_last,ss_ (ng/mL·h)	1775; (445.3–2875)	1.187; (6.236–7522)	0.67; (0.54–0.84)

AUC_last_: area under the curve from 48 h to 120 h at steady-state; CI: confidence interval; C_max,ss_: maximum drug concentration in plasma at steady-state.

**Table 5:** Maximum simulated concentrations at steady-state for tramadol and *O*-desmethyltramadol (M1) in each organ (C_max,ss_) after oral administration of 100 mg tramadol ER tablet twice daily (five times in total).

Organ	Maximum Concentration at Steady-State in Each Organ (ng/mL)	
	Tramadol	M1
Adipose tissue	400.6	12.89
Bone	899.8	23.88
Brain	1034	19.74
Gut	2741	88.38
Heart	763.0	94.97
Kidney	1452	78.46
Liver	3034	157.5
Lung	991.0	21.09
Muscle	2411	79.13
Pancreas	2000	56.25
Skin	1355	42.03
Spleen	2821	88.53

**Table 6:** Predicted geometric mean C_max,ss_ and AUC_last,ss_ values for tramadol and *O*-desmethyltramadol (M1) following oral administration of 100 and 200 mg tramadol ER tablet twice daily (total five times) in various CYP2D6 metabolizer groups.

Parameters	UM		EM		IM		PM	
	Tramadol	M1	Tramadol	M1	Tramadol	M1	Tramadol	M1
***Tramadol 100 mg***								
C_max,ss_ (ng/mL) (range)	357.2 (72.43–927.6)	126.8 (21.39–449.0)	469.6 (122.2–1117)	83.80 (12.62–368.1)	593.8 (165.8–1379)	40.93 (5.511–240.9)	721.3 (209.5–1675)	0.6433 (0.0975–5.312)
AUC_last,ss_ (ng/mL·h) (range)	5353 (648.7–25,267)	1881 (278.6–8560)	8206 (1217–34,213)	1445 (175.2–7522)	12,049 (1932–42,462)	813.5 (79.54–5234)	16,795 (2682–61,319)	2.919 (0.3351–34.42)
***Tramadol 200 mg***								
C_max,ss_ (ng/mL) (range)	714.3 (144.9–1855)	253.5 ^#^ (42.77–898.0)	939.1 * (244.3–2235)	167.6 (25.24–736.3)	1188 ** (331.7–2758)	81.86 (11.02–481.7)	1443 ** (418.9–3349)	1.287 (0.1950–10.62)
AUC_last,ss_ (ng/mL·h) (range)	10,706 (1297–50,533)	3761 (557.1–17,119)	16,411 (2434–68,426)	2890 (350.3–15,044)	24,097 (3864–84,923)	1627 (159.1–10,467)	33,591 (5365–122,637)	5.839 (0.67–68.83)

AUC_last,ss_: area under the curve from 48 h to 120 h at steady-state; C_max,ss_: maximum drug concentration in plasma at steady-state; EM: extensive metabolizer; IM: intermediate metabolizer; PM: poor metabolizer; UM: ultra-rapid metabolizer. * Above the therapeutic range for tramadol (>800 ng/mL); ** in toxic range for tramadol (1000–2000 ng/mL); ^#^ above maximum therapeutic range for M1 (>200 ng/mL).

**Table 7:** Predicted and observed geometric mean PK parameters for tramadol and M1 according to CYP2D6 genotype following oral administration of 100 mg tramadol ER tablet twice daily (total five times).

**Tramadol**	**EM**			**IM**			**PM**		
	**Observed** **(*n* = 13)**	**Predicted** **(*n* = 1000)**	**Ratio** **(90% CI)**	**Observed** **(*n* = 8)**	**Predicted** **(*n* = 1000)**	**Ratio** **(90% CI)**	**Observed** **(*n* = 1)**	**Predicted** **(*n* = 1000)**	**Ratio** **(90% CI)**
C_max,ss_ (ng/mL) (range)	551.2 (294.0–904.4)	469.6 (122.2–1117)	0.85 (0.72–1.01)	828.5 (676.6–942.1)	593.8 (165.8–1379)	0.72 (0.59–0.87)	751.10	721.3 (209.5–1675)	0.96
AUC_last,ss_ (ng/mL·h) (range)	7116 (4127–9345)	8206 (1217–34,213)	1.15 (0.90–1.48)	13,501 (10,527–16,038)	12,049 (1932–42,462)	0.89 (0.66–1.20)	8591.72	16,795 (2682–61,319)	1.95
**M1**	**EM**			**IM**			**PM**		
	**Observed** **(*n* = 13)**	**Predicted** **(*n* = 1000)**	**Ratio** **(90% CI)**	**Observed** **(*n* = 8)**	**Predicted** **(*n* = 1000)**	**Ratio** **(90% CI)**	**Observed** **(*n* = 1)**	**Predicted** **(*n* = 1000)**	**Ratio** **(90% CI)**
C_max,ss_ (ng/mL) (range)	125.0 (81.8–176.7)	83.80 (12.62–368.1)	0.67 (0.52–0.86)	87.79 (66.0–114.1)	40.93 (5.511–240.9)	0.47 (0.32–0.69)	29.8	0.6433 (0.0975–5.312)	0.02
AUC_last,ss_ (ng/mL·h) (range)	1996 (1373–2875)	1445 (175.2–7522)	0.72 (0.56–0.94)	1718 (1223–2199)	813.5 (79.54–5234)	0.47 (0.31–0.72)	445.3	2.919 (0.3351–34.42)	0.01

AUC_last,ss_: area under the curve from 48 h to 120 h at steady-state; CI: confidence interval; C_max,ss_: maximum drug concentration in plasma at steady-state; EM: extensive metabolizer; IM: intermediate metabolizer; PM: poor metabolizer; Ratio = predicted/observed. Since the observed data for the PM group are for 1 subject, the CI value cannot be obtained.

## Supplementary Materials

The following are available online at https://www.mdpi.com/1999-4923/11/11/618/s1, Figure S1: The predicted mean tramadol and *O*-desmethyltramadol concentration-time profiles after administration of 100 mg tramadol ER tablet twice daily (five times in total) for CYP2D6 poor metabolizer, intermediate metabolizer, extensive metabolizer and ultra-rapid metabolizer, respectively; Figure S2: The predicted mean tramadol and *O*-desmethyltramadol concentration-time profiles after administration of 200 mg tramadol ER tablet twice daily (five times in total) for CYP2D6 poor metabolizer, intermediate metabolizer, extensive metabolizer and ultra-rapid metabolizer, respectively.
