# Supplementary material for: Evaluation of the Effect of CYP2D6 Genotypes on Tramadol and O-Desmethyltramadol Pharmacokinetic Profiles in a Korean Population Using Physiologically-Based Pharmacokinetic Modeling
Source: Pharmaceutics. 2019 Nov 17;11(11):618. doi: 10.3390/pharmaceutics11110618 (PMC6920759; doi:10.3390/pharmaceutics11110618)
Supplement: Supplementary file 1 [file pharmaceutics-11-00618-s001.pdf]

pharmaceutics

Supplementary Materials: Evaluation of the Effect of CYP2D6 Genotypes on Tramadol and *O*-Desmethyltramadol Pharmacokinetic Profiles in a Korean Population Using Physiologically-Based Pharmacokinetic Modeling

Hyeon-Cheol Jeong, Soo Hyeon Bae, Jung-Woo Bae, Sooyeun Lee, Anhye Kim, Yoojeong Jang and Kwang-Hee Shin

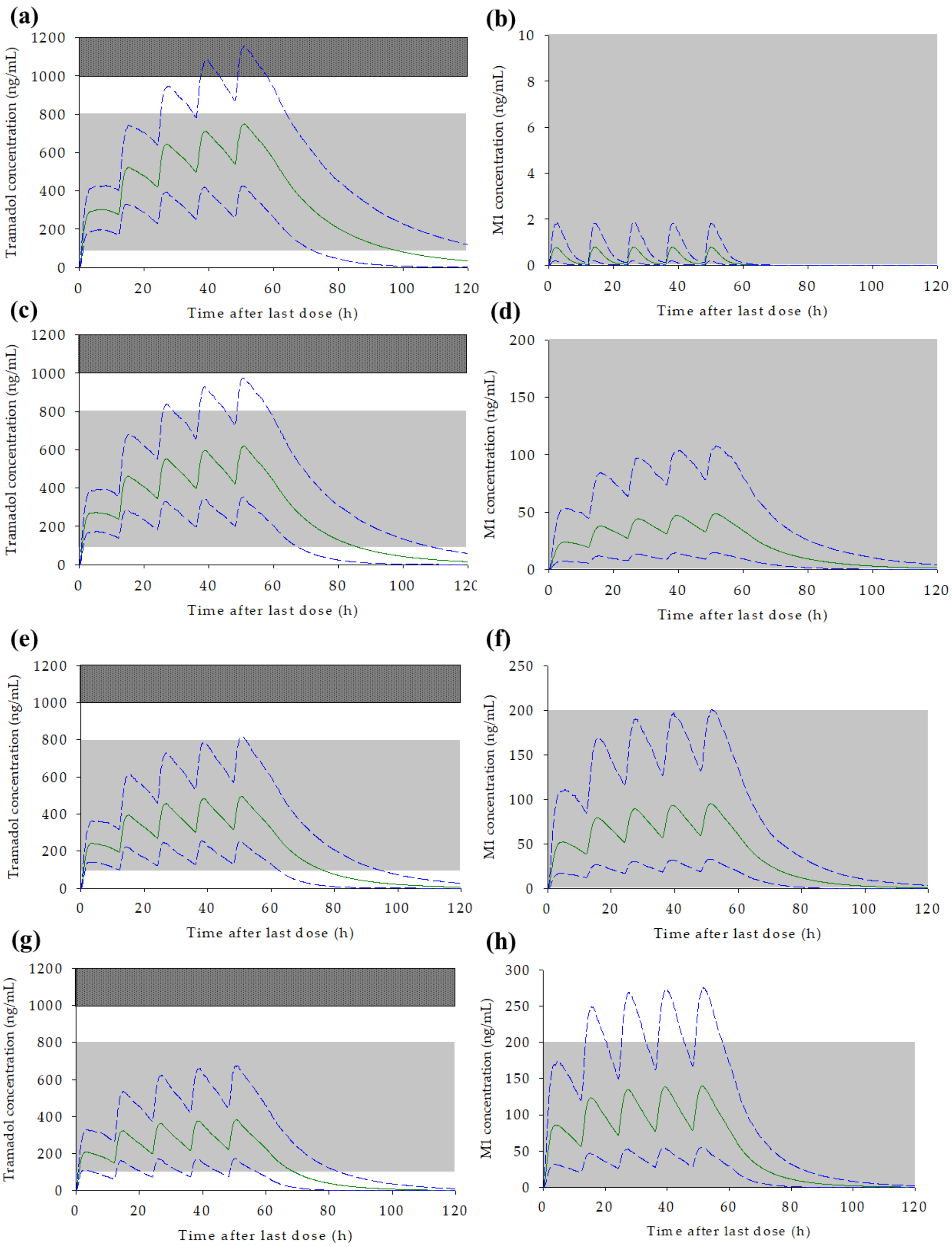

Figure S1. The predicted mean tramadol and *O*-desmethyltramadol concentration-time profiles after administration of 100 mg tramadol ER tablet twice daily (five times in total) for CYP2D6 poor metabolizer (**a** and **b**), intermediate metabolizer (**c** and **d**), extensive metabolizer (**e** and **f**) and ultra-rapid metabolizer (**g** and **h**), respectively. Blue dashed lines represent 95th and 5th percentile ranges, and dark green solid line represents predicted mean concentration. Gray areas and checked gray areas in (**a**), (**c**), (**e**), and (**g**) represent the therapeutic concentration range (100–800 ng/mL) and the toxic range (above 1000 ng/mL) for tramadol, respectively; and the gray area in (**b**), (**d**), (**f**), and (**h**) represents the maximum therapeutic range for M1 (up to 200 ng/mL).

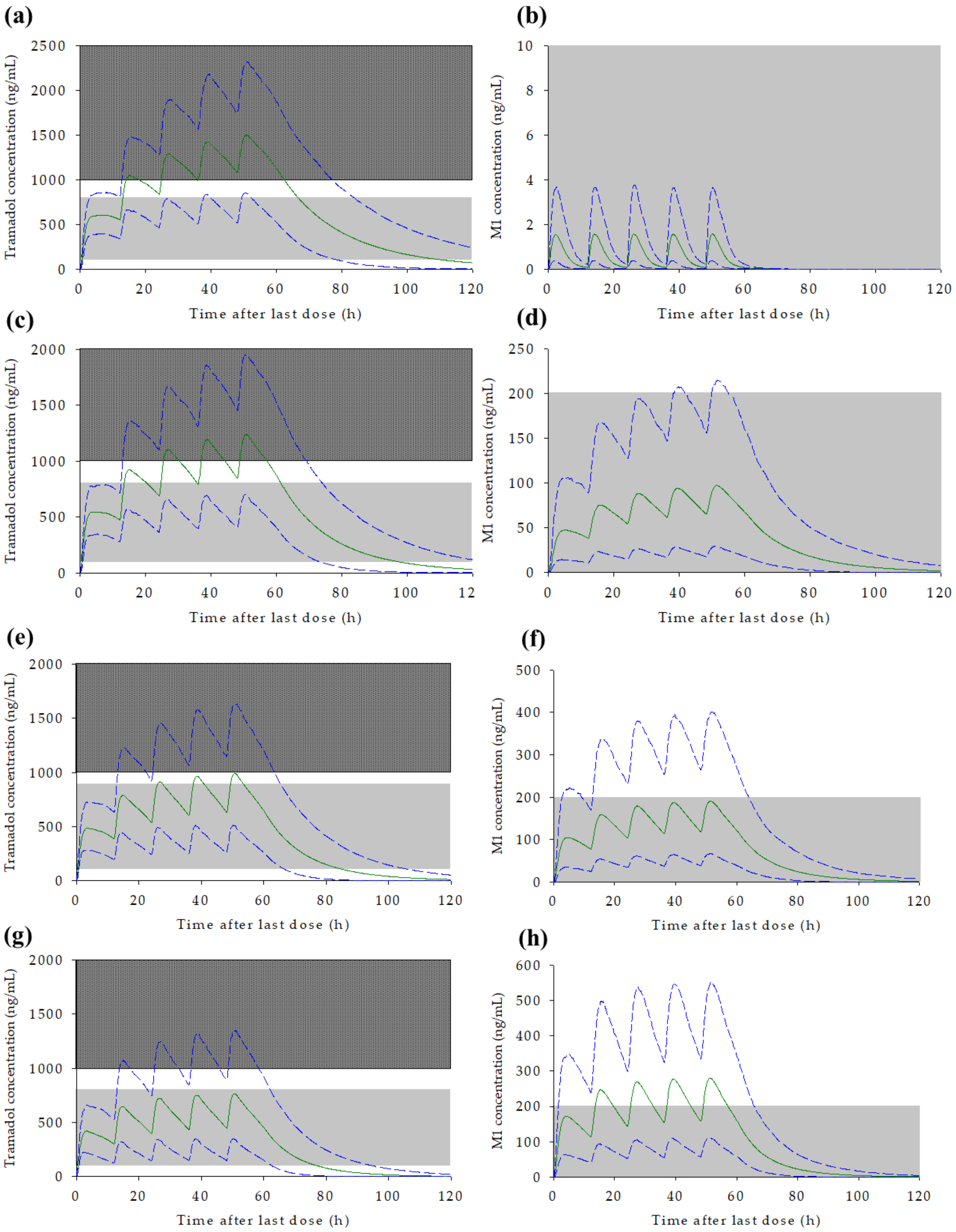

Figure S2. The predicted mean tramadol and *O*-desmethyltramadol concentration-time profiles after administration of 200 mg tramadol ER tablet twice daily (five times in total) for CYP2D6 poor metabolizer (**a** and **b**), intermediate metabolizer (**c** and **d**), extensive metabolizer (**e** and **f**) and ultra-rapid metabolizer (**g** and **h**), respectively. Blue dashed lines represent 95th and 5th percentile ranges, and dark green solid line represents predicted mean concentration. Gray areas and checked gray areas in (**a**), (**c**), (**e**), and (**g**) represent the therapeutic concentration range (100–800 ng/mL) and the toxic range (above 1000 ng/mL) for tramadol, respectively; and the gray area in (**b**), (**d**), (**f**), and (**h**) represents the maximum therapeutic range for M1 (up to 200 ng/mL).
